# 免疫检查点抑制剂相关血液毒性处理的临床诊疗建议

**DOI:** 10.3779/j.issn.1009-3419.2019.10.13

**Published:** 2019-10-20

**Authors:** 俊玲 庄, 静婷 赵, 潇潇 郭, 佳鑫 周, 炼 段, 维 邱, 晓燕 斯, 丽 张, 玥 李, 小伟 刘, 汉萍 王, 道斌 周, 力 张

**Affiliations:** 1 100730 北京，中国医学科学院，北京协和医院血液科 Department of Hemotology, Peking Union Medical College Hospital, Chinese Academy of Medical Sciences, Beijing 100730, China; 2 100730 北京，中国医学科学院，北京协和医院心内科 Department of Cardiology, Peking Union Medical College Hospital, Chinese Academy of Medical Sciences, Beijing 100730, China; 3 100730 北京，中国医学科学院，北京协和医院风湿免疫科 Department of Rheumatology and Immunology, Peking Union Medical College Hospital, Chinese Academy of Medical Sciences, Beijing 100730, China; 4 100730 北京，中国医学科学院，北京协和医院内分泌科 Department of Endocrinology, Peking Union Medical College Hospital, Chinese Academy of Medical Sciences, Beijing 100730, China; 5 100730 北京，中国医学科学院，北京协和医院肾内科 Department of Nephrology, Peking Union Medical College Hospital, Chinese Academy of Medical Sciences, Beijing 100730, China; 6 100730 北京，中国医学科学院，北京协和医院呼吸内科 Department of Respirology, Peking Union Medical College Hospital, Chinese Academy of Medical Sciences, Beijing 100730, China; 7 100730 北京，中国医学科学院，北京协和医院检验科 Department of Clinical Laboratory, Peking Union Medical College Hospital, Chinese Academy of Medical Sciences, Beijing 100730, China; 8 100730 北京，中国医学科学院，北京协和医院消化内科 Department of Gastroenterology, Peking Union Medical College Hospital, Chinese Academy of Medical Sciences, Beijing 100730, China; 9 100730 北京，中国医学科学院，北京协和医院眼科 Department of Ophthalmology, Peking Union Medical College Hospital, Chinese Academy of Medical Sciences, Beijing 100730, China

**Keywords:** 免疫检查点抑制剂, 免疫相关不良反应, 血细胞减少, Immune checkpoint inhibitor, Immune-related adverse events, Cytopenia

## Abstract

免疫检查点抑制剂能够重新激活免疫系统，启动肿瘤杀伤，而T细胞过度激活导致各种免疫相关不良反应。血液系统不良反应报道少见，主要表现为单系或多系血细胞减少，包括自身免疫性溶血性贫血、免疫性血小板减少症、中性粒细胞减少症，严重时表现为再生障碍性贫血，甚至可能致命，如噬血细胞淋巴组织细胞增多症。我们将总结免疫检查点抑制剂相关血液不良反应的临床特点，并对诊断和治疗给予建议。

## Abstract (en)

Immune checkpoint inhibitors are able to reactivate the immune system therefore enhance the anti-tumor effects. However, over-activated T cells may induce immune related adverse events (irAEs). Hematological irAEs are rarely reported, which mainly represent as mono-lineage cytopenia or pancytopenia, including autoimmune hemolytic anemia (AIHA), immune thrombocytopenia (ITP), neutropenia and aplastic anemia, sometimes even lethal, such as hemophagocytic lymphohistiocytosis. The clinical manifestations of hematological irAEs will be summarized and recommendations of diagnosis and treatment are proposed.

2018年诺贝尔医学奖授予在肿瘤免疫治疗领域获得杰出成绩的两位科学家，其实关于肿瘤免疫治疗的探索已经进行了几十年。尤其是过去十年间我们对肿瘤和免疫系统相互作用的认识飞速发展。肿瘤细胞激活免疫检查点（immuno-checkpoints），例如分子程序性死亡受体-1（molecular programmed death receptor-1, PD-1）和细胞毒性T淋巴细胞相关抗原4（cytotoxic T lymphocyte associated antigen 4, CTLA-4）等信号通路，抑制T淋巴细胞活化，达到免疫逃逸，即“免疫刹车”的作用^[1]^。而免疫检查点抑制剂（immuno-checkpoint inhibitors, ICIs）则通过抑制CTL-4或PD-1，解除免疫抑制、活化T淋巴细胞，从而恢复对肿瘤细胞的清除。因此，免疫治疗也成为许多种肿瘤治疗的新策略。然而，由此产生的免疫相关的不良事件（immune-related adverse effects, irAEs）也逐渐引起关注^[2]^。这些irAEs主要包括免疫器官、皮肤、肺脏、内分泌、消化系统等，而血液相关AEs报道甚少，主要包括血细胞减少、噬血细胞淋巴组织细胞增多症（hemophagocytic lymphohistiocytosis, HLH）等，一旦发生，需要暂时终止抗肿瘤治疗，有些甚至可能危及生命。本文将对ICIs相关血液AEs进行总结并给出初步诊疗建议。

ICIs引起的血液AE罕见，统计了9, 324例患者的荟萃分析显示贫血、粒细胞减少症和血小板减少症发生率分别为9.8%、0.94%和2.8%^[3]^。可以单独表现为一系下降，也可以表现为两系或全血细胞减少，例如血小板减少合并中性粒细胞减少，甚至再生障碍性贫血。具体机制尚无研究，主要考虑为irAEs。具体包括以下几方面内容。

## 1 自身免疫性溶血性贫血（autoimmune hemolytic anemia, AIHA）

AIHA是报道最多的血液AE，截至目前共有68例病例报告，男女比例相当。基础疾病主要是黑色素瘤（41%）和非小细胞肺癌（non-small cell lung cancer, NSCLC）（26%），其他包括肾癌、霍奇金淋巴瘤或皮肤肿瘤等。报道的病例主要来自北美（49%）和欧洲（34%），少数为亚洲（10%）和澳大利亚（7%）。60%的患者为单独应用抗PD-1单抗，而且绝大部分是nivolumab；19%为抗CTLA-4，16%为二者合用，还有4%为抗PD-L1单抗^[4]^。

临床表现：AIHA发生的时间大多集中在用药60 d之内（中位时间50 d），也有少数超过180 d的报道。其中4例同时发生血小板减少，4例合并内分泌异常，包括甲状腺异常、肾上腺功能低减或垂体炎等，还有3例合并消化道AE，如结肠炎或肝炎等。实验室检测均有Coombs试验阳性，多数为IgG型，少数为单纯C3型。多数为温抗体型，极少数为冷抗体型^[4]^。

诊断和鉴别诊断：由于肿瘤进展或其他因素也可导致贫血，因此需要鉴别其他原因导致的贫血，包括检查血清铁和铁蛋白、血清叶酸、维生素B12等排除营养性贫血。确诊指标主要包括网织红细胞计数升高，间接胆红素升高，乳酸脱氢酶升高以及Coombs试验阳性。骨髓检查并非必需，为了排查其他原因导致的贫血，例如骨髓异常增生综合征、肿瘤骨髓侵犯或合并罕见的纯红细胞再生障碍性贫血（pure red cell aplastic, PRCA）时需要进行骨髓穿刺和活检。

治疗：超过80%的患者贫血达到重度，即 < 60 g/L，需要输血支持，个别病例因为延迟诊断，严重贫血导致器官衰竭，相关死亡率约15%。一经诊断，所有患者均接受了激素治疗，建议泼尼松初始剂量为1.5 mg/kg -2 mg/kg，2周-4周后开始减量，治疗持续时间3个月左右。有的患者在过早停用激素后AIHA复发，再次应用仍有效。尽管AIHA整体呈现良性过程，应用激素期间需停用ICIs治疗^[4-10]^。

是否能在AIHA控制后再次给予ICIs治疗目前尚无定论，有报道1例霍奇金淋巴瘤患者应用2剂nivolumab疗效肯定，因AIHA停药，激素治疗3个月溶血缓解。再次应用nivolumab 5次未再发生AIHA^[10]^。因此，可以根据患者疗效和AIHA是否控制决定能否再次应用ICIs。

## 2 免疫性血小板减少症（immune thrombocytopenia, ITP）

第二位发生率最高的血液学AE为ITP。由于肿瘤患者出现血小板减少比较常见，而ITP并无特征性诊断指标，因此，即使在应用ICIs之后发生也需排查其他病因，例如感染、肿瘤进展或其他药物相关。目前也无ITP的明确易感因素，应用ICIs之前免疫疾病可能增加ITP发生率^[11]^。

在报道的57例ICIs相关ITP中，最多的仍是黑色素瘤和NSCLC，报道的病例主要来自北美（53%）和亚洲（33%），少数为欧洲（14%）。65%的患者为单独应用抗PD-1单抗；16%为抗CTLA-4，18%为二者合用，还有2%为抗PD-L1单抗^[4]^。

临床表现：大多数ITP发生时间在用药后12周之内，中位时间约41 d。其中7例合并消化系统AE，4例合并其他血液AE，另外还有少数合并皮肤和内分泌异常病例。严重程度分级和其他血小板（platelet, PLT）减少相同，1级为PLT计数在75, 000个/μL-100, 000个/μL；2级计数在50, 000个/μL -75, 000个/μL之间时；3级降至25, 000个/μL-50, 000个/μL；4级为 < 25, 000个/μL。通常达到4级才会出现严重自发出血，包括皮肤黏膜出血等，严重的可以危及生命^[4, 5]^。

诊断和鉴别诊断：除血常规、外周血细胞涂片外，自身抗体检测阳性为ITP诊断的佐证，但并非必需。尽管骨髓检查对原发性ITP并不要求，但对irAEs来说，需要进行骨髓涂片细胞学和活检，以排除血小板减少是骨髓增生不良还是外周性破坏过多。ITP大多表现为骨髓巨核细胞增多，或者基本正常，且以未成熟巨核细胞为主，即颗粒巨核细胞。另外需要排除感染相关PLT减少，包括细菌培养（临床怀疑）、Epstein-Barr病毒（Epstein-Barr virus, EBV）、巨细胞病毒（cytomegalovirus, CMV）、细小病毒B19、肝炎病毒、人类免疫缺陷病毒（human immunodeficiency virus, HIV）等。

治疗：发生1级PLT减少或PLT计数低于基线数值50%即需要密切监测，至少每周两次，尚不需停用ICIs药物。2级PLT减少即需暂停ICIs药物，短暂应用皮质激素2周-4周再逐渐减量。3级-4级减少时除停用免疫治疗外，尚需尽快开始泼尼松1 mg/kg-2 mg/kg，必要时应用静脉注射用免疫球蛋白（intravenous immune globulin, IVIG），这些与成人原发性ITP的处理类似。如果PLT快速反应，激素可以在足量应用2周-4周后逐渐减停。一线治疗失败，可以考虑利妥昔单抗、脾切除术、血小板受体激动剂或二线免疫抑制药物如环孢素、硫唑嘌呤等^[4, 5, 11-13]^。

虽然1级-2级血小板减少在短暂或永久停用ICIs药物后可能自行恢复，最近发表的荟萃分析显示78%的ITP为4级减少，所有患者均应用激素治疗，2/3同时应用了IVIG，22%患者对治疗并无反应，因此换用血小板生成素受体激动剂或利妥昔单抗。ITP缓解后是否再次应用ICIs尚无定论，3例患者再次应用后有1例再发血小板减少，因此需平衡再次用药的风险和获益^[4, 5, 11]^。

## 3 中性粒细胞减少症

中性粒细胞减少症是一种罕见的irAEs，最多的荟萃分析有9例。其中ipilimumab最多见，nivolumab，pembrolizumab其次。病种主要为黑色素瘤、NSCLC和前列腺癌。由于ICIs治疗相关的中性粒细胞减少症缺乏特征性诊断指标，因此需要排查其他病因，包括病毒感染、其他化疗药物等，如果是单药应用ICIs，则可以排除其他化疗药物的因素^[5]^。

临床表现：中性粒细胞减少症发生的中位时间为用药后第3周期，均为3级-4级减少。20%-30%患者合并出现其他irAEs，包括皮疹、消化道反应和其他血液学异常等。粒细胞减少会继发细菌或真菌感染，因此这些患者常常出现发热，需要应用抗生素治疗^[5]^。

治疗：由于所有粒细胞减少均为3级-4级，一旦发生，所有患者均停用ICIs，而且没有粒细胞恢复后再次应用的报道。所有患者均应用了皮质激素和粒细胞集落刺激因子（granulocyte colony stimulating factor, G-CSF），粒细胞约在治疗2周左右恢复正常。尽管激素增加感染风险，但单纯应用G-CSF是否足够尚无定论。1例患者同时合并贫血和血小板减少，除激素和G-CSF外还应用了霉酚酸酯，环孢菌素A和抗胸腺细胞球蛋白（antithymocyte globulin, ATG）进行治疗。另有1例患者粒细胞恢复后并未再次应用ICIs，而中性粒细胞减少在缓解8周后再发，应用同样治疗后再次恢复^[14-20]^。

## 4 再生障碍性贫血（aplastic anemia, AA）和纯红细胞再生障碍性贫血（pure red cell aplasia, PRCA）

两系或三系血细胞减少发生机制与单系减少相似，为异常表达的T细胞受体分子，包括免疫检查点CTLA-4和PD-1下调引起免疫应答过度激活，CD4/CD8比例倒置提示抑制性T细胞致敏。这与普通AA或PRCA有相似之处。

最多的荟萃分析报道AA+PRCA共有17例患者，中位年龄分别为59岁和65岁。病种主要为黑色素瘤，单独应用抗PD-1抗体的共有10例。同样，引起全血细胞减少的原因众多，而且AA本身也是排除性诊断，因此需要排查细菌/病毒感染、其他药物等因素^[4, 5]^。

临床表现和鉴别诊断：AA/PRCA通常发生在应用ICIs约10周，与其他血液AE并无太多不同。中性粒细胞减少可以达到3级-4级，贫血从2级-4级不等。尚有PRCA合并AIHA的报道。粒细胞严重下降会导致相关感染风险增加，严重贫血可能带来心脏等器官供血不足，4级血小板减少可能造成严重出血。如出现发热，细菌和病毒指标需要检查，考虑PRCA时尤其需要检查细小病毒B19抗体或核酸。建议进行骨髓细胞学和病理活检，一方面准确了解骨髓增生情况，一方面排除肿瘤浸润等其他原因。AA的骨髓造血细胞全部呈现低减，通常未见巨核细胞；PRCA红系造血细胞比例低于5%。可能出现抗核抗体（antinuclear antibody, ANA）高滴度阳性，因此更需要进行骨髓检查以排除自身免疫病相关的全血细胞减少^[4, 5, 21-23]^。

治疗：即使感染风险增加，所有患者也均接受了皮质激素和细胞因子支持治疗，包括G-CSF和血小板生成素受体激动剂等，同时输血、输血小板、抗感染。尽管所有患者均停用ICIs，而且未再次应用，AA/PRCA的疗效并不像单一血细胞减少的结局那样乐观。17例中仅有3例完全恢复，有1例因粒细胞缺乏伴发热最终死于感染。两系下降的患者似乎恢复起来稍快^[4, 5, 21-23]^。

尽管目前缺乏巡证结果，我们建议出现全血细胞减少时给予预防性抗感染药物，包括抗病毒、真菌等，同时应用泼尼松或甲泼尼松龙1 mg/kg，同时应用G-CSF和输血支持，如2周后仍无恢复，建议尽快加用环孢素3 mg/kg/d-5 mg/kg/d±雄激素并减停激素。必要时再应用ATG。

## 5 噬血细胞淋巴组织细胞增多症（hemophagocytic lymphohistiocytosis, HLH）

HLH是炎症因子大量激活导致多器官衰竭的一种罕见综合征，进展快，死亡率极高，且临床表现不特异，与重症感染、肿瘤进展有很多相似之处，因此高死亡率部分归因于诊断延误。ICIs相关HLH与T细胞异常激活有关^[24]^。

荟萃分析中最大宗的报道是26例HLH，多为黑色素瘤患者，CTLA-4抑制剂较PD1/PD-L1单抗更为多见（58% *vs* 34%; *P*=0.02）。HLH发生更早，在应用ICIs后中位26 d发生，相关死亡率也是所有血液irAEs中最高的，达到23%^[4]^。

临床表现和诊断/鉴别诊断：诊断HLH参照2004标准，具体包括：①发热；②脾大；③2系-3系外周血细胞减少；④高甘油三酯血症（≥265 mg/dL）和/或低纤维蛋白原血症（≤1.5 g/L）；⑤骨髓/脾脏/淋巴结出现噬血现象；⑥NK细胞活性降低；⑦血清铁蛋白≥500 μg/L；⑧可溶性CD25（sIL-2受体）≥2, 400 U/mL。符合5/8条或以上即可以诊断HLH。其中铁蛋白超过10, 000 μg/L对HLH诊断的敏感性为90%，特异性为96%。sCD25水平对诊断疾病严重程度有帮助^[24]^。

尚有19%患者合并EBV感染，说明免疫反应放大后激活了潜在的感染。EBV或其他感染可以触发HLH，因此需要进行细菌培养或其他病毒检测以排查其他引起HLH的病因。

治疗：ICIs相关HLH并无确定指南可以遵循，均参照国际组织细胞学会的HLH-94和2004方案，即以大剂量地塞米松为主，联合依托泊苷或环孢素等。中国尚有联合脂质体多柔比星，依托泊苷和甲泼尼松龙作为成人难治性HLH患者的挽救治疗^[25]^。就目前报道来看，多数患者仅应用大剂量泼尼松或甲泼尼松龙1 mg/kg-2 mg/kg，另有个别患者联合应用霉酚酸酯或利妥昔单抗^[26-29]^。

建议以大剂量激素为主，参照HLH国际方案联合依托泊苷，效果不佳再考虑生物治疗（如利妥昔单抗、英夫利昔单抗和依那西普）和抗白细胞介素-6（托珠单抗）等。

## 6 结语

ICIs相关的血液系统不良反应主要表现为一系或多系血细胞减少，严重者可危及生命。目前尚无标准化指南参考，一旦出现，建议进行血液和骨髓检查，并与感染、其他药物作用等鉴别。治疗上以大剂量激素为主，必要时加用其他免疫抑制剂，同时做好感染防控和支持治疗。
